# Impact of a Tailored Nutrition and Lifestyle Intervention for Overweight Cancer Survivors on Dietary Patterns, Physical Activity, Quality of Life, and Cardiometabolic Profiles

**DOI:** 10.1155/2019/1503195

**Published:** 2019-11-21

**Authors:** Colleen K. Spees, Ashlea C. Braun, Emily B. Hill, Elizabeth M. Grainger, James Portner, Gregory S. Young, Matthew D. Kleinhenz, Chureeporn Chitchumroonchokchai, Steven K. Clinton

**Affiliations:** ^1^Medical Dietetics & Health Sciences, School of Health and Rehabilitation Sciences, The Ohio State University College of Medicine, Columbus, Ohio, USA; ^2^Comprehensive Cancer Center, The Ohio State University, Columbus, Ohio, USA; ^3^Department of Internal Medicine, Division of Medical Oncology, The Ohio State University, Columbus, Ohio, USA; ^4^College of Social Work, The Ohio State University, Columbus, Ohio, USA; ^5^Center for Biostatistics, The Ohio State University, Columbus, Ohio, USA; ^6^Department of Horticulture and Crop Science, The Ohio State University, Columbus, Ohio, USA

## Abstract

Survivors of cancer often experience treatment-related toxicity in addition to being at risk of cancer recurrence, second primary cancers, and greater all-cause mortality. The objective of this study was to test the safety and efficacy of an intensive evidence-based garden intervention to improve outcomes for cancer survivors after curative therapy. To do so, a clinical trial of adult overweight and obese cancer survivors within 2 years of completing curative therapy was completed. The 6-month intervention, delivered within the context of harvesting at an urban garden, combined group education with cooking demonstrations, remote motivational interviewing, and online digital resources. Data on dietary patterns, program satisfaction, and quality of life were collected via questionnaires; anthropometrics, physical activity, and clinical biomarkers were measured objectively. Of the 29 participants, 86% were white, 83% were female, and the mean age was 58 years. Compared to baseline, participants had significant improvements in Healthy Eating Index (HEI) scores (+5.2 points, *p* = 0.006), physical activity (+1,208 steps, *p* = 0.033), and quality of life (+16.07 points, *p* = 0.004). Significant improvements were also documented in weight (−3.9 kg), waist circumference (−5.5 cm), BMI (−1.5 kg/m^2^), systolic BP (−9.5 mmHg), plasma carotenoids (+35%), total cholesterol (−6%), triglycerides (−14%), hs-CRP (−28%), and IGFBP-3 (−5%) (all *p* < 0.010). These findings demonstrate a tailored multifaceted garden-based biobehavioral intervention for overweight and obese cancer survivors after curative therapy is safe and highly effective, warranting larger randomized controlled trials to identify program benefits, optimal maintenance strategies, program value relative to cost, and approaches for integration into a survivor's oncology management program. This trial is registered on ClinicalTrials.gov NCT02268188.

## 1. Introduction

Advances in cancer diagnosis and treatment have led to a greater proportion of patients achieving a complete remission and durable cure [1]. However, survivors face a multitude of short- and long-term physical and mental health comorbidities, in addition to those present at diagnosis [2]. Moreover, over 60% of cancer survivors in the U.S. are considered overweight or obese, increasing the risk of additional cancers and sequelae of metabolic syndrome, reduced physical functioning, and all-cause mortality [3, 4]. Cancer survivors also experience health issues secondary to the rigors of cancer therapy associated with surgical interventions, radiation, and chemotherapeutics, disrupting nutritional status, physical function, and metabolism [1].

We currently lack evidence from studies integrating diet, nutrition, and physical activity for cancer survivors demonstrating long-term improvement in health outcomes [5, 6]. Thus, standard of care nutrition and physical activity programs are not routinely integrated into oncology care, a problem compounded by financial barriers, such as lack of coverage by insurance programs. Organizations including the World Cancer Research Fund/American Institute for Cancer Research (WCRF/AICR) have formulated evidence-based cancer prevention guidelines focused on maintaining a healthy body weight, physical fitness, and a primarily plant-based dietary pattern [7, 8]. Expert committees and clinicians advise cancer survivors to follow public health guidelines (e.g., the Dietary Guidelines for Americans and the WCRF/AICR recommendations) in the absence of more precise programs [7–11].

Theory-driven approaches addressing multiple lifestyle behaviors in cancer survivors have demonstrated promise, yet there remains a need to develop and evaluate a more effective intervention, both in the proportion of individuals responding and the degree of change, than current standards and define biobehavioral strategies that enhance outcomes and promote maintenance [12]. More recently, a greater appreciation exists for individual variation in response to behavioral programs, and future success depends upon the development of tailored interventions. Increasingly, reviews of relevant interventions to improve health outcomes have concluded standard, formulaic approaches show modest short-term efficacy, but poor long-term success [13, 14]. The ongoing challenge is to define, implement, and evaluate tailored lifestyle programs targeting high-risk subgroups such as those with overweight or obesity, amongst others.

We have previously developed an integrated intervention strategy, and the current study presents an adaptation to this strategy to target overweight and obese cancer survivors. Described in detail elsewhere, our comprehensive, theory-driven intervention combines group and tailored individual education coupled with an enriched environment in hopes of promoting social stimulation, group support, behavior change, and self-management to elicit a significant response in cardiometabolic outcomes and quality of life (QOL) [15]. Evidence in human and animal models highlights the potential for cognitive and social stimulation in low-stakes settings to promote beneficial psychological effects, including modulation of neuroplasticity, particularly those that are natural versus manufactured (e.g., characterized by vegetation) [16, 17]. Furthermore, active participation in gardening has been documented to increase the quantity and variety of produce consumed while increasing physical activity and functional status comparably to other moderate-intensity activities [18–20]. Increased consumption of produce has been linked to displacement of calories and energy-dense foods, which can contribute to weight loss [21]. Preliminary studies suggest garden-based interventions targeting those with nutrition-related chronic disease demonstrate improved adherence as compared with nongarden-based approaches, yet few garden studies have specifically targeted overweight and obese cancer survivors [15, 22, 23].

Aligning with the evidence-based guidelines, our tailored intervention was designed to achieve higher compliance than past dietary and fitness interventions for cancer survivors and improvements in metrics of health and QOL and dietary and physical activity patterns [23–25]. Our intensive intervention was previously evaluated to assess feasibility and preliminary efficacy prior to proceeding to the development of a larger randomized intervention compared with standards of care [15]. In the current study, our objective was to determine the safety and efficacy of the intervention after adapting it to meet the unique needs of overweight and obese cancer survivors. We hypothesized our intervention would be successfully adapted to this population of survivors and demonstrate safety and efficacy.

## 2. Materials and Methods

### 2.1. Participants

Recruitment was conducted in local oncology clinics, community centers, and other communication channels targeting survivors. Participants were English-speaking survivors (≥18 years) who completed active cancer treatment within the previous 48 months (current adjuvant hormone therapy was acceptable), currently without evidence of active cancer, and a body mass index (BMI) ≥25 kg/m^2^. Participants were ineligible if they were cognitively unable to consent; participating in recent or ongoing diet and exercise programs; diagnosed with conditions precluding physical activity; consuming medications with dietary contraindications (e.g., warfarin); unwilling to discontinue nonprescribed supplements, herbals, or botanicals; diagnosed with significant metabolic or digestive disorders, renal or hepatic insufficiency, cachexia, and short bowel syndrome; or pregnant [15]. All study procedures were approved by the Institutional Review Board at The Ohio State University. All participants provided written informed consent.

### 2.2. Intervention

This trial was designed to assess the safety and efficacy of a novel and intensive multifaceted lifestyle intervention for overweight or obese adult cancer survivors. Informed by a feasibility pilot in nonobese survivors, we further refined and expanded our program to integrate tailored components, including multiple biobehavioral tools with documented value in previous work for specialized support [14, 15, 26]. A greater emphasis was placed on theoretical aspects for education to encourage autonomy in selecting avenues for improving adherence to cancer prevention guidelines, including group and one-on-one interactions addressing knowledge gaps while facilitating the transformation of targeted information to tailored goals [14, 26, 27]. In brief, the 6-month intervention components included (1) weekly urban garden experiences and harvesting (fruits, vegetables, and herbs) [28]; (2) semimonthly group education classes, each including a 30-minute interactive discussion surrounding evidence-based guidelines; (3) semimonthly cooking demonstrations, complementing group education and using produce harvested from the study garden to encourage incorporation into meals and to provide opportunities for taste testing [7, 8, 29, 30]; (4) remote motivational interviewing coaching (tele-MI) [31]; and (5) supportive technologies, including a pedometer to track steps and access to a secure web portal with multiple functions.

Collectively, the intervention was designed to encourage achievement of numerous participant-level goals; throughout, registered dietitians (RDs) promoted personal goal setting and empowerment to adopt and sustain positive behavior change. A primary objective of the program was to foster the adoption of a primarily plant-based dietary pattern in order to displace calories from energy-dense sources. Participants were encouraged to follow recommendations from the 2015-2020 U.S. Dietary Guidelines for Americans as well as those specifically outlined in cancer survivor-specific recommendations and achieve a daily 500-calorie deficit [7–9]. In addition, participants were encouraged to achieve the recommendations set forth by the Physical Activity Guidelines for Americans (e.g., 150 minutes of moderate-intensity exercise per week, or 10,000 steps per day); these two goals combined to contribute to a weight loss of approximately one pound per week [32, 33].

#### 2.2.1. Garden Experience

A key principle of this intervention was the integration of an enriched environment into the program with the goal of enhancing the efficacy and impact on physical and mental health outcomes. Accordingly, our integrated and enriched garden-based intervention included harvesting fresh garden produce one to three times per week. This intervention component offered participants the opportunity to derive both cognitive and social stimulation. To further optimize the enriched environment, the study components were offered in a supportive and relaxed manner, allowing for flexibility in participation of some components (e.g., optional tele-MI).

The garden was a 3-acre plot integrated within a 261-acre agricultural research farm on the university campus, staffed for this study with RDs and horticulture students. The garden was planted to offer a wide variety of produce over the growing season. Adjacent to the garden is an indoor university classroom equipped for cooking demonstrations. At enrollment, participants were oriented to farm policies, safety issues, and harvesting techniques and provided a harvest bag and registration card to electronically track participation.

#### 2.2.2. Group Education

Study participants attended faculty-guided group education sessions every two weeks. Each 30-minute session focused on at least one of the evidence-based recommendations, including those relating to dietary and physical activity patterns, and was facilitated by a local expert and included an interactive question and answer session [7, 9]. While delivering targeted information for cancer survivors, these sessions served to provide participants information they needed to encourage and empower them to adopt a diet consistent with evidence-based guidelines, including displacement of calories by shifting to a primarily plant-based dietary pattern.

#### 2.2.3. Cooking Demonstrations

To encourage utilization of garden produce and provide opportunities for skill development, each group education session was coupled with an interactive cooking demonstration provided by a medical center chef and RD. Lasting approximately 30 minutes, each demonstration included descriptions and hands-on examples to reinforce basic cooking and food preparation techniques (e.g., knife skills). Recipes prepared incorporated available produce from the study garden to empower participants to utilize these skills to prepare similar meals and snacks at home.

#### 2.2.4. Motivational Interviewing

In order to provide tailored support while maximizing intrinsic motivation, an RD served as the tele-MI coach for this intervention after appropriate training [31]. Well-defined and implemented MI has proven efficacious in various settings and populations [34, 35]. MI has been implemented in interventions utilizing remote platforms as a mechanism for behavior change; thus, methods for interacting with the tele-MI coach in this intervention were chosen by each participant (e.g., email, text, and telephone) during the course of the study [36]. In brief, each participant was contacted within one week of their baseline assessment, with weekly contacts attempted thereafter. Each correspondence was based on individual goals and served to assist participants in addressing barriers, overcoming ambivalence, or otherwise supporting individual needs; all tele-MI interactions remained separate from study-related reminders. The MI methodology has been previously reported [31].

#### 2.2.5. Supportive Technologies

To supplement the educational material covered in the education classes, participants were provided with access to a secure web portal which housed cancer survivor-specific information, additional resources, and electronic copies of handouts and recipes from classes. Updated weekly, the website housed links to external websites and information on the benefits of the available garden produce. Participants were encouraged to utilize the website when missing a class or if more information was required. Participants were also encouraged to weigh themselves and submit logs to the secure web portal weekly. This remote tracking was reviewed by the tele-MI coach, who created individual and de-identified group graphical records depicting change over time, allowing participants to view individual and group progress to stimulate continued participation. The tele-MI coach also provided additional information, including links to educational materials and evidence-based resources.

The overarching goal of our intervention was to achieve greater compliance with the evidence-based guidelines coupled with greater impact on biomarkers than previous dietary and fitness interventions for cancer survivors [7, 9]. Therefore, our objectives were to improve participant (1) weight; (2) dietary patterns; (3) physical activity patterns; (4) QOL; and (5) relevant biomarkers of health.

### 2.3. Data Collection

#### 2.3.1. Dietary Patterns

Participants at baseline and postintervention reported their consumption of food and beverages over 30 days using the VioScreen Graphical Food Frequency Questionnaire (FFQ, Viocare, Inc., Princeton, NJ). This algorithm-driven, computer-delivered FFQ uses the Nutrition Data System for Research database (Nutrition Coordinating Center, University of Minnesota) for analysis. Diet quality was assessed using Healthy Eating Index 2010 (HEI), which measures compliance with the U.S. Dietary Guidelines for Americans [37]. These scores were automatically tabulated by VioScreen utilizing previously described methods [38].

#### 2.3.2. Physical Activity Patterns

Given low baseline levels, our physical activity goal was modest, focusing on daily steps. Participants received pedometers (Omron Healthcare Co. Inc., Lake Forest, IL) which served to motivate and reinforce behavior change, as well as for data collection. Participants reviewed their daily steps and uploaded numbers weekly to the secure web portal. The tele-MI coach also reviewed these data and provided graphical tracking records to show change over time.

#### 2.3.3. Clinical and Laboratory Measurements

Participants completed laboratory visits at baseline and postintervention following a 12-hour fast and 72-hour period of avoidance of vigorous exercise or alcohol consumption. Visits were conducted between 7:00 am and 10:00 am. Participants were weighed wearing light clothing and no shoes on a calibrated Pro Plus digital scale (Health-o-Meter Professional Products, Pelstar LLC, Bridgeview, IL) to the nearest 0.1 kg. Height was measured using a calibrated stadiometer (Health-o-Meter Professional Products, Pelstar LLC, Bridgeview, IL) to the nearest 1 mm. Three waist circumference (WC) measurements were obtained between the costal margin and the iliac crest to the nearest 1 mm. Blood pressure was obtained using an Omron Autocuff (Omron Healthcare Co. Ltd., Lake Forest, IL) standardized against a manual sphygmomanometer. Skin carotenoid levels were assessed noninvasively with a Pharmanex Nu Skin BioPhotonic Scanner S3 (Nu Skin Enterprises, Provo, UT), utilizing resonance Raman spectroscopy [39].

Venous blood samples were obtained by trained phlebotomists with 20 mL of blood into Vacutainer® tubes (Becton, Dickinson and Co., Franklin Lakes, NJ). EDTA Vacutainer tubes were used for carotenoid profiling by a high performance liquid chromatography-diode array detector following previously developed methods [40]. Blood samples were immediately processed for lipid profiles, hemoglobin A1c (HbA1c), adiponectin, insulin, leptin, and inflammatory markers hs-CRP, IGF-1, and IGFBP-3 using standard protocols.

#### 2.3.4. Quality of Life and Other Behaviors

The Quality of Life Patient/Cancer Survivor Version (QOL-CSV) [41] questionnaire was used to estimate perceived health including physical well-being, psychological well-being, social concerns, and spiritual well-being. Additional personal, health, and behavioral information were collected via modified Behavioral Risk Factor Surveillance System (BRFSS) questions and additional questions to assess motivation [42, 43]. Self-efficacy was assessed via the New General Self-Efficacy scale and additional study-specific questions to assess participant confidence in adhering to evidence-based guidelines [44].

#### 2.3.5. Program Evaluation

Following completion of the intervention, participants were provided a comprehensive questionnaire to provide feedback on the program, including each of its components. These include closed- and open-ended questions to elicit both quantitative indicators of participants' perceptions as well as qualitative data.

### 2.4. Statistical Analyses

Statistical analyses for the effect of the intervention on anthropometric, dietary, and clinical measures compared the baseline and postintervention values, testing the null hypothesis of no change in these variables using paired *t*-tests. Values for lipids, inflammatory markers, and plasma carotenoids were log transformed prior to analysis due to heteroscedasticity. For these outcomes, differences from baseline to postintervention were expressed as fold-change. For evaluation of compliance, participations were considered compliant if they attended/utilized ≥75% of the in-person education sessions and/or related remote components. All analyses were performed in SPSS version 23 (SPSS Inc, Chicago, IL) or SAS v9.4 (SAS Institute, Cary, NC).

## 3. Results

### 3.1. Recruitment, Retention, and Baseline Characteristics

A total of 56 adult cancer survivors were screened for eligibility, and 35 (*n* = 28 female, *n* = 7 male) were deemed eligible and enrolled. During the study, 2 were removed due to cancer recurrence, 1 withdrew due to a noncancer health issue, and 3 due to personal issues. No grade 3 or 4 adverse events were documented based upon Common Terminology Criteria for Adverse Events (v4.0). Of the final cohort (*n* = 29), the majority were white and female (86.2% and 82.8%, respectively, Table 1). The mean age was 58.0 years, and the mean age of initial cancer diagnosis was 52.9 years for females and 65.2 years for males. Breast (44.8% of total, 54.2% of females) and prostate (17.2% of total, 100% of males) cancers were the most prevalent primary cancers.

### 3.2. Attendance and Adherence

Compliance with each of the multiple components of the intervention was high. On average, participants attended 90% (9/10) of the education sessions, and mean class attendance was 24 of 29 participants (84%). Individually, participants attended 15 of 25 weeks of harvest (59%), with greater attendance on weeks when education sessions were scheduled. All participants submitted pedometer steps, with 15 of 29 participants (52%) completing every week of the 6-month intervention, while 26 of 29 (90%) submitted step data for at least 80% of the weeks. Twenty-six participants (90%) reported use of the secure web portal, and 59% of participants utilized tele-MI. For individual communication, participants requested use of email (*n* = 14, 48.3%), phone (*n* = 3, 10.3%), text message (*n* = 2, 6.9%), or mixed preferences/no preference (*n* = 10, 34.5%). In total, 71% of interactions occurred via e-mail while 57% of participants used telephonic interactions and 10% used text messages, described in more detail elsewhere [31].

### 3.3. Dietary and Physical Activity Patterns

Participants improved their adherence to the dietary and physical activity evidence-based guidelines for cancer survivorship. Aligning with weight loss goals, participants demonstrated improvements in measures of dietary intakes, including a decrease in daily mean energy intake (−250 kcal, *p* = 0.012), an increase in vegetable and fruit consumption (+1.05 servings, *p* < 0.001 and +0.41 servings, *p* = 0.022, respectively), and a decrease in consumption of added sugars (−2.37 tsp., *p* = 0.036) from baseline to postintervention (data not presented here). Increases in diet quality based upon significant improvements of HEI scores from baseline to postintervention are shown in Table 2. Total diet scores improved by 5.2 points on a 100-point scale (*p* = 0.006). Scores for total fruit (+0.8, *p* = 0.003), whole fruit (+0.6, *p* = 0.009), fatty acids (+1.5, *p* = 0.007), refined grains (+1.1, *p* = 0.013), and empty calories (+2.1, *p* = 0.008) also improved; scores for total vegetables trended positively (+0.4, *p* = 0.054).

Carotenoid status, which served as a biomarker of produce intake, increased from pre- to postintervention. Total dietary carotenoid intakes increased by 66% (*p* < 0.001), including increases in individual intakes of all 5 major carotenoids consumed in the diet (data not presented here). Likewise, total plasma carotenoids improved significantly (+35%, *p* < 0.001, Table 3) as did several individual carotenoids (e.g., alpha-carotene *p* < 0.001, total beta-carotene *p* < 0.001, total lycopene *p*=0.017). Skin carotenoids also increased over the course of the intervention (*p*=0.015) and demonstrated a strong, positive correlation with total plasma carotenoids (*r* = 0.73, *p* < 0.001).

Compared with baseline, participants increased their physical activity patterns to more closely align with evidence-based guidelines. Indeed, mean steps per day increased from 6,560 to 7,768 (+18.9%, *p*=0.033) over the course of the intervention.

### 3.4. Anthropometric and Clinical Measures

Changes in anthropometric measures and clinical indicators are detailed in Table 3. Significant reductions were noted in body weight (−3.9 kg), BMI (−1.5 kg/m^2^), WC (−5.5 cm), total cholesterol (TC) (−6%), systolic BP (−9.5 mmHg), and triglycerides (TG) (−14%). Analysis of inflammatory markers revealed significant decreases in hs-CRP and IGFBP-3 (by 28%, *p*=0.004 and 5%, *p*=0.005, respectively) as well as decreases in insulin levels (by 3%), though these failed to reach significance. Participant logged weights demonstrate consistent moderate weight loss throughout the intervention (Supplementary [Supplementary-material supplementary-material-1]).

### 3.5. Qualitative Measures

Overall QOL significantly improved (+16.07 points, *p*=0.004) as well as several subscales indicating improvements in physical, psychological, and spiritual well-being, characterized by fewer feelings of distress secondary to illness and treatment, as well as cancer-related fears (Table 4). Positive trends were noted in total self-efficacy (+3%, *p*=0.061), with stronger findings amongst study-specific items (i.e., adherence to cancer prevention guidelines; data not presented here). Participants reported taking fewer medications and supplements at postintervention (prescribed and over-the-counter), with fewer challenges associated with eating healthy. Specifically, fewer participants described barriers related to cost, dislike of healthy food, knowledge regarding preparation and what constitutes healthy food, access, desire, ease of purchase, and willpower.

### 3.6. Acceptability of Intervention

Participants reported high acceptability of the intervention (Table 5). Ninety-three percent rated both the program and harvesting as “excellent” or “very good.” Amongst all components, participants rated the group education sessions most effective (55%), followed by harvesting (34%) and tele-MI (18% of those that utilized). Participants reported the program impacted their overall health in a positive manner (97%), provided them with a sense of community and support (93%), and stated they would recommend the program to other survivors (97%). Twenty-eight participants (97%) agreed the program helped them achieve better dietary patterns, and 27 participants (93%) agreed the program helped them improve their physical activity patterns. Based on program evaluation, all participants (100%, *n* = 29) planned to use the information gleaned to make future health-related decisions.

## 4. Discussion

Obesity, metabolic syndrome, declines in physical fitness, and their sequelae are common in cancer survivors [1]. Coupled with additional risks of chronic toxicities and complications from cancer therapies, it is imperative survivors have access to safe and effective interventions to reverse these risks and promote heath and QOL. To date, while numerous interventions have been designed for cancer survivors and have shown modest success, tailored programs with individualized support are few and further are not standardized or fully integrated into cancer care, similar to cardiac rehabilitation models [12]. The development of such programs and data to demonstrate the safety, efficacy, and value (cost/impact) derived from well-designed and rigorous clinical trials is critical. Those programs showing promise can move forward into randomized multi-institutional studies in comparison with the current standards of care, which are minimal.

The objective of this study was to evaluate the safety and efficacy of our multicomponent intervention adapted to a population of overweight and obese cancer survivors, with the overarching goal of this intervention being improved adherence to the evidence-based guidelines. The recruitment goals were met, and the present cohort was similar with respect to age, sex, and sociodemographic profiles to those in comparable lifestyle interventions for cancer survivors, and the cancer diagnoses of those recruited were similar to those frequently targeted [45]; our cohort included a higher percentage of females due to the strong breast cancer program at our institution. The retention and participation was higher than previously reported studies of diet/lifestyle interventions in cancer survivors [46]. Indeed, attrition from the trial was just over 15%, with the major issues for withdrawal or removal being travel, vacations, and disease recurrence. Safety of the intervention is supported by the absence of grade 3 and 4 adverse events based upon Common Terminology Criteria for Adverse Events (v4.0). Participation in the key components of the intervention, including lectures and cooking demonstrations (84% attendance), garden harvesting (84% during weeks with class, 59% on off-weeks), pedometer utilization (90%), web-based utilization (90%), and tele-MI (59%) was high. We attribute such high retention and participation rates to the tailored and flexible approach coupled with the intensity and frequency of contact, leading to greater improvements in key outcomes compared to those reported elsewhere [47].

Participants indicated the program helped them better align their lifestyle behaviors with the evidence-based guidelines for cancer survivorship, a finding which was collectively reinforced by positive outcomes in self-report measures, objective indices of health, and clinical biomarkers. Assessments of dietary patterns indicated participants more closely aligned with a plant-based dietary pattern at postintervention [7–9]. This improvement is potentially consequential if sustained, as recent analyses of national cancer survivor outcomes demonstrated high diet quality is associated with a substantial reduction in overall and cancer-specific mortality, leading authors to conclude that high-quality diets may protect against death among survivors [48, 49]. In addition to improved intakes of specific food groups, the study cohort's HEI improvement of >5 points translates into an estimated 5% decrease in mortality when compared to a similarly aged population [50]. It is important to note the total HEI score for the study cohort was 22 points higher at baseline than the total HEI score documented for cancer survivors across the U.S. [51]. This finding is likely due to healthier survivors being more likely to commit to a diet and exercise program coupled with higher income, greater formal education, and Caucasian race, all characteristics of trial participants [52]. Nonetheless, these findings were mirrored by increases in skin and plasma carotenoids, which serve as a quantitative biomarker associated with reported produce intakes [39, 53]. The increased plasma concentrations of multiple individual carotenoids demonstrated increased consumption of a variety of fruits and vegetables [53]. Evidence that dietary patterns high in fruits and vegetables and modest in intakes of sodium, added sugar, and saturated fat are inversely related to cardiovascular disease, and all-cause mortality informs the guidelines for cancer survivors [7, 54]. These dietary changes are reflective of displacement of energy-dense, nutrient-depleted, and highly processed foods for nutrient- and phytochemical-rich options, which also contributes to calorie reductions.

Other clinical biomarkers, including inflammatory indicators and those related to cardiometabolic health, also improved after the intervention, consistent with other behavioral interventions promoting primarily plant-based dietary patterns [55]. The present study resulted in significant improvements in weight, BMI, WC, systolic BP, TC, TG, and hs-CRP. Specifically, participants achieved a sustainable weight loss of approximately 0.25 kg (>0.5 pounds) per week over the 24 weeks of the study in parallel with increases in physical activity by nearly 20%. Furthermore, WC decreased 5%, translating into a 9% decreased risk of all-cause mortality and decrease in cardiovascular disease risk [56, 57]. In participants who were obese at baseline, the intentional weight loss is associated with a 15% decrease in all-cause mortality risk and is further associated with a reduction in cardiometabolic risk in cancer survivors [58]. Leptin and adiponectin trended positively, though these measures generally demonstrate great variability among individuals and our study lacked sufficient power to detect significant changes. These data, taken together, support the conclusion that this intervention, based upon energy balance, dietary patterns, and fitness, has the potential if sustained to have significant cardioprotective benefits.

A recent systematic review documents the emerging literature regarding garden-based interventions, indicating changes in beliefs, knowledge, and attitudes surrounding healthy food, as well as improvements in healthy food practices (e.g., variety of produce consumed), while highlighting the need for assessment of objective biomarkers of health [59]. Emerging thematic patterns suggest potential value of integration of enriched environment experiences within strategies for behavior change. While challenging to measure, the data presented here demonstrate this, with participants reporting significant improvements in QOL and indices of health, including physical, psychological, and spiritual well-being. In comparison, studies of cancer survivors document a decrease in general QOL, including measures of mental and physical well-being, social functioning, vitality, pain, and capacities to fulfill physical and emotional roles one to two years after diagnosis [60]. The high compliance in participation and impact on measured outcomes is likely, in part, due to the social networking that occurs with the shared garden experience [61]. Indeed, 93% reported both a sense of community and an overall positive impression of the garden experience. We can speculate the instructive and natural environment may have contributed to improvements in biomarkers of health. Evidence in animal models has demonstrated such natural environments can similarly elicit improvements in emotional health and neurobiological responses contributing to behavioral modifications, such as shifts in motivation [17]. It is plausible the combination of a mentored garden experience with hands-on learning activities (e.g., cooking demonstrations) may have rendered similar benefits. Coupled with cooking demonstrations, the garden experience effectively introduced new varieties of produce, altered previous taste preferences, encouraged adventurous eating, modified perceptions regarding cost and availability, and taught new preparation techniques.

Our experience suggests that MI can contribute to individual success by emphasizing autonomy, addressing ambivalence, and promoting intrinsic motivation through ongoing one-on-one support. In line with previous behavioral research, the incorporation of MI as one component of a multicomponent lifestyle intervention can promote long-term changes in health outcomes, including weight loss, improved dietary patterns, and increases in physical activity, in both the general population and in cancer survivors [34, 35, 62, 63]. Results from program evaluations indicated that 18% of tele-MI users perceived it as the most effective component of the intervention. While not ranked as the most effective intervention component overall, these results suggest that for a percentage of participants, MI is beneficial. We believe that a multifaceted intervention which encourages participants to engage in various components is empowering and promotes success. In this cohort, greater weight loss (4.8 vs. 2.6 kg) and improvements in QOL (*p*=0.030), amongst other variables, were observed with utilization of tele-MI [31].

We report a multidisciplinary and highly integrated garden-based intervention that significantly improved dietary and physical activity patterns, as well as clinical and laboratory markers of health in overweight and obese cancer survivors. The program employs multiple tools for tailoring the intervention for individuals, integrating feedback and support mechanisms, and promoting behavior change through evidence-based core curriculum, all of which is provided to cancer survivors in a low-pressure enriched garden environment. As a culmination of theory-driven techniques previously shown to result in clinically relevant outcomes as well as those capable of improving QOL, these components work in concert to provide flexibility while enhancing intrinsic motivation, commitment to change, and overall well-being [64, 65]. Our strategy going forward is to continue to integrate a portfolio of options during an intervention suited to individual needs, based upon unique life schedules, computer/technical savvy, education and backgrounds, and comorbidities, yet all integrated within a uniform evidence-based dietary and fitness program.

Although caution remains in the interpretation of a single-arm study, the main value is demonstrating an intervention with high retention and impact. Thus, randomized, large-scale studies are critical to test the ability to implement this effort and compare with standards of care. Limitations include the absence of a control group and the small size and homogeneity of the final cohort, all of which limit the generalizability of our results. The few participants who withdrew from the study speak to limitations secondary to participants' inability to maintain attendance in the context of personal challenges, such as transportation. Amongst those who completed the intervention and in whom positive results were documented, there is the possibility these advantageous outcomes were the result of the Hawthorne effect. In addition, the “healthy participant” effect may be present, in which participants report healthier behaviors than nonparticipants. This may contribute to higher baseline values (e.g., HEI); however, the documented improvement over time challenges this, as well as concomitant improvement in biological values, which have previously shown to correlate to self-reported outcomes [15]. The results of this work warrant continued research to elucidate the relationship between psychological and biological outcomes.

## 5. Conclusions

We have demonstrated high compliance and impact of a multifaceted but fully integrated and tailored program targeting dietary and physical activity patterns, weight, and cardiometabolic outcomes in cancer survivors. Most critically, the inclusion of tele-MI and a garden experience likely contributed significantly to the improvement of multiple quantified outcomes, including QOL. To better assess this impact, future studies must emphasize long-term maintenance. In parallel, large-scale studies comparing this program to standards of care, including evaluation of the costs and potential benefits for cancer survivors as well as future healthcare utilization, are necessary. These key studies may allow for a cancer survivor program to be fully integrated into cancer care similarly to cardiac rehabilitation strategies, now considered standard of care and reimbursed by payers.

## Figures and Tables

**Table 1:** Demographics and characteristics of overweight cancer survivors participating in a 6-month behavioral intervention study (*n* = 29).

	Participant characteristics	Valid %^a^ (*n*)
Age (years)		58.0

Sex	Female	82.8 (24)
	Male	17.2 (5)

Race/ethnicity	White/Caucasian	86.2 (25)
	Black/African American	10.3 (3)
	Asian	3.4 (1)

Marital status	Married	62.1 (18)
	Divorced	13.8 (4)
	Never married	13.8 (4)
	Others^b^	10.3 (3)

Education	Less than grade 12/grade 12 equivalent	10.3 (3)
	College 1 to 3 years	10.3 (3)
	College 4 years or more	44.8 (13)
	Professional or graduate	34.5 (10)

Employment	Employed or self-employed	51.7 (15)
	Retired	44.8 (13)
	Out of work < 1 year	3.4 (1)

Household income	>$50,000	51.7 (15)
	$10,000–$49,999	27.6 (8)
	Prefer not to answer/do not know	20.7 (6)

Primary cancer diagnosis (age, years)	Female	52.9
	Male	65.2

Primary cancer	Breast	44.8 (13)
	Prostate	17.2 (5)
	Ovarian/uterine	13.8 (4)
	Colorectal	6.9 (2)
	Others^c^	17.3 (5)

Data are presented as % and *n* and include baseline characteristics of participants that completed both baseline and postintervention data collection visits. ^a^Percentage based upon the number of participants for whom data was available. ^b^Widowed, separated, member of an unmarried couple, or prefer not to answer. ^c^Lymphoma (10.3%), brain (3.4%), and pancreatic (3.4%).

**Table 2:** Change in Healthy Eating Index scores in overweight cancer survivors participating in a 6-month behavioral intervention study (*n* = 29).

HEI component	Max score	Baseline	Postintervention	Mean difference (95% CI)	Unadjusted *p* value
*Adequacy (higher score indicates higher consumption)*					
Total diet	100	69.6 ± 12.3	74.8 ± 9.8	+5.2 (1.6, 8.8)	0.006^*∗*^
Total fruit^a^	5	3.6 ± 1.5	4.4 ± 1.1	+0.8 (0.3, 1.3)	0.003^*∗*^
Whole fruit^b^	5	4.2 ± 1.3	4.8 ± 0.7	+0.6 (0.2, 1.0)	0.009^*∗*^
Total vegetables^c^	5	4.5 ± 0.9	4.9 ± 0.4	+0.4 (−0.01, 0.7)	0.054
Greens and beans^c^	5	4.3 ± 1.2	4.6 ± 1.0	+0.3 (−0.2, 0.7)	0.307
Whole grains	10	5.9 ± 3.6	5.7 ± 3.5	−0.2 (−1.2, 0.7)	0.607
Dairy^d^	10	7.5 ± 2.3	6.8 ± 2.9	−0.7 (−1.6, 0.1)	0.082
Total protein foods^e^	5	4.7 ± 0.5	4.4 ± 1.0	−0.3 (−0.6, 0.1)	0.170
Seafood and plant proteins^e,f^	5	4.3 ± 1.0	4.5 ± 0.9	+0.2 (−0.1, 0.5)	0.148
Fatty acids^g^	10	4.7 ± 3.0	6.2 ± 3.2	+1.5 (0.5, 2.6)	0.007^*∗*^

*Moderation (higher score indicates lower consumption)*					
Refined grains	10	8.7 ± 2.2	9.8 ± 0.7	+1.1 (0.3, 2.0)	0.013^*∗*^
Sodium	10	2.7 ± 2.8	2.3 ± 2.9	−0.4 (−1.7, 0.8)	0.449
Empty calories^h^	20	14.4 ± 4.3	16.5 ± 3.9	+2.1 (0.6, 3.6)	0.008^*∗*^

Data are presented as mean ± standard deviation, and changes are expressed as mean differences for participants that completed both baseline and postintervention assessment visits. Data are Healthy Eating Index 2010 (HEI) scores for participants that completed both baseline and postintervention assessments. HEI is a scoring metric that assesses diet quality as specified by the US Dietary Guidelines for Americans [37]. It is made up of 12 components: 9 for adequacy and 3 for moderation. A higher score indicates better conformance to dietary guidance, and the total HEI score is the sum of the component scores. HEI, Healthy Eating Index. ^a^Includes 100% fruit juice. ^b^Includes all forms except juice. ^c^Includes any beans and peas not counted toward total protein foods. ^d^Includes all milk products, such as fluid milk, yogurt, and cheese, and fortified soy beverages. ^e^Beans and peas are included here (not with vegetables) when the total protein foods standard is otherwise not met. ^f^Includes seafood, nuts, seeds, and soy products (other than beverages) as well as beans and peas counted as total protein foods. ^g^Ratio of poly- and monounsaturated fatty acids (PUFAs and MUFAs) to saturated fatty acids (SFAs). ^h^Calories from solid fats, alcohol, and added sugars; threshold for counting alcohol is >28 g/day. ^*∗*^*p* < 0.05.

**Table 3:** Change in anthropometric and clinical biomarkers in overweight cancer survivors participating in a 6-month behavioral intervention study (*n* = 29).

Variable	Baseline	Postintervention	Mean difference or fold change (95% CI)	Unadjusted *p* value
Weight (kg)	85.3 ± 16.2	81.4 ± 16.7	−3.9 (−5.6, −2.2)	<0.001^*∗*^
Body mass index (kg/m^2^)	31.9 ± 5.1	30.4 ± 5.3	−1.5 (−2.1, −0.8)	<0.001^*∗*^
Waist circumference (cm)	102.0 ± 13.6	96.5 ± 13.6	−5.5 (−6.9, −4.1)	<0.001^*∗*^
Systolic BP (mmHg)	127.7 ± 15.8	118.1 ± 13.0	−9.5 (−16.0, −3.0)	0.006^*∗*^
Diastolic BP (mmHg)	75.0 ± 8.3	73.2 ± 8.0	−1.8 (−4.7, 1.0)	0.197
HbA1c (%)	5.7 ± 0.5	5.7 ± 0.5	0.0 (−0.3, 0.3)	0.879
Total cholesterol (mg/dL)^a^	190.4 ± 29.5	179.2 ± 32.2	0.94 (0.90, 0.98)	0.004^*∗*^
HDL (mg/dL)^a^	54.9 ± 13.3	53.4 ± 13.0	0.97 (0.92, 1.03)	0.275
LDL (mg/dL)^a^	113.5 ± 28.6	107.7 ± 29.0	0.95 (0.89, 1.00)	0.052
Triglycerides (mg/dL)^a^	133.2 ± 52.7	113.1 ± 44.4	0.86 (0.76, 0.96)	0.010^*∗*^
hs-CRP (mg/L)^a^	4.0 ± 4.2	3.3 ± 4.1	0.72 (0.58, 0.89)	0.004^*∗*^
IGFBP-3 (*μ*g/mL)^a^	4.7 ± 0.9	4.5 ± 0.8	0.95 (0.91, 0.98)	0.005^*∗*^
IGF-1 (ng/mL)^a^	95.6 ± 34.1	104.5 ± 40.1	1.07 (0.86, 1.33)	0.553
Leptin (ng/mL)^a^	35.7 ± 33.0	29.4 ± 28.7	0.71 (0.40, 1.26)	0.226
Adiponectin (*µ*g/mL)^a^	12.9 ± 87.3	13.0 ± 71.9	1.06 (0.76, 1.47)	0.740
Insulin (pg/mL)^a^	490.7 ± 310.9	459.6 ± 253.2	0.97 (0.72, 1.30)	0.821
Total skin carotenoids (RRS counts)	29,509 ± 11,471	33,963 ± 14,441	4,455 (944, 7,965)	0.015^*∗*^
Total plasma carotenoids (nmoL/L)^a^	1,749.5 ± 871.7	2,330.0 ± 1220.8	1.35 (1.15, 1.58)	<0.001^*∗*^
Lutein + zeaxanthin (nmoL/L)^a,b^	98.7 ± 60.0	125.3 ± 84.1	1.27 (0.98, 1.64)	0.066
Beta-cryptoxanthin (nmoL/L)^a^	143.71 ± 162.4	121.1 ± 76.1	1.02 (0.82, 1.27)	0.840
Alpha-carotene (nmoL/L)^a^	140.7 ± 95.2	293.5 ± 263.6	1.91 (1.52, 2.40)	<0.001^*∗*^
Beta-carotene all-trans (nmoL/L)^a^	603.8 ± 507.1	884.5 ± 676.0	1.56 (1.22, 2.01)	0.001^*∗*^
Beta-carotene–cis (nmoL/L)^a^	65.7 ± 38.9	78.2 ± 39.1	1.29 (1.03, 1.62)	0.028^*∗*^
Total beta-carotene (nmoL/L)^a^	669.5 ± 539.3	962.7 ± 711.8	1.50 (1.21, 1.86)	<0.001^*∗*^
Lycopene all-trans (nmoL/L)^a^	526.7 ± 257.5	601.9 ± 244.6	1.20 (0.99, 1.44)	0.060
Lycopene–cis (nmoL/L)^a^	189.5 ± 116.1	247.7 ± 112.4	1.46 (1.12, 1.89)	0.006^*∗*^
Total lycopene (nmoL/L)^a^	716.2 ± 361.9	849.6 ± 336.7	1.26 (1.05, 1.53)	0.017^*∗*^

Data are presented as mean ± standard deviation, and changes are expressed as mean differences or fold change for participants that completed both baseline and postintervention assessment visits. BP, blood pressure; HbA1c, hemoglobin A1c; HDL, high-density lipoprotein; LDL, low-density lipoprotein; hs-CRP, high-sensitivity C-reactive protein; IGFBP-3, insulin-like growth factor-binding protein-3; IGF-1, insulin-like growth factor 1. ^a^Log transformed prior to analysis and difference expressed as fold change. ^b^All plasma zeaxanthin values below detectable limit. ^*∗*^*p* < 0.05.

**Table 4:** Change in select quality of life scores in overweight cancer survivors participating in a 6-month behavioral intervention study (*n* = 29).

Item	Baseline	Postintervention	Mean difference (95% CI)	Unadjusted *p* value
Quality of life (total score)	268.86 ± 51.24	284.93 ± 51.75	+16.07 (5.5, 26.6)	0.004^*∗*^

*Physical well-being*				
Fatigue	5.59 ± 2.64	6.52 ± 2.72	+0.93 (0.01, 1.86)	0.049^*∗*^
Appetite changes	7.93 ± 2.42	8.14 ± 2.25	+0.21 (−0.76, 1.17)	0.664
Sleep changes	6.79 ± 2.57	7.17 ± 2.35	+0.38 (−0.60, 1.36)	0.436
Constipation	8.52 ± 2.25	8.07 ± 2.51	−0.45 (−1.19, 0.29)	0.223
Please rate your overall physical health	6.24 ± 2.12	7.10 ± 1.47	+0.86 (−0.05, 1.77)	0.062

*Psychological well-being*				
How good is your quality of life?	7.17 ± 2.27	8.28 ± 1.22	+1.10 (0.18, 2.03)	0.021^*∗*^
How much happiness do you feel?	7.59 ± 1.52	7.79 ± 1.47	+0.21 (−0.33, 0.74)	0.432
Do you feel like you are in control of things in your life?	7.10 ± 2.04	7.45 ± 1.76	+0.34 (−0.31, 1.00)	0.289
How satisfying is your life?	7.48 ± 1.84	7.86 ± 1.43	+0.38 (−0.17, 0.93)	0.170
How useful do you feel?	7.21 ± 2.14	7.76 ± 1.81	+0.55 (0.06, 1.05)	0.030^*∗*^

*To what extent are you fearful of*:				
Future diagnostic tests	5.38 ± 3.11	6.45 ± 2.43	+1.07 (0.06, 2.08)	0.039^*∗*^
A second cancer	5.72 ± 2.93	6.66 ± 2.91	+0.93 (−0.06, 1.92)	0.064
Recurrence of your cancer	4.79 ± 3.06	5.83 ± 3.35	+1.03 (0.09, 1.98)	0.033^*∗*^
Spreading (metastasis) of your cancer	5.31 ± 3.42	6.48 ± 3.29	+1.17 (0.16, 2.18)	0.025^*∗*^

*Social concerns*				
Is the amount of support you receive from others sufficient to meet your needs?	8.00 ± 2.55	7.79 ± 2.51	−0.21 (−0.96, 0.55)	0.580
To what degree has your illness and treatment interfered with your employment?	6.93 ± 3.34	8.59 ± 2.13	+1.66 (0.66, 2.65)	0.002^*∗*^
How much isolation do you feel is caused by your illness or treatment?	8.00 ± 2.58	8.10 ± 2.76	+0.10 (−0.45, 0.65)	0.703

*Spiritual well-being*				
How much has your spiritual life changed as a result of your cancer diagnosis?	5.17 ± 3.35	5.93 ± 3.23	+0.76 (−0.61, 2.13)	0.266
To what extent has your illness made positive changes in your life?	5.72 ± 2.76	6.48 ± 2.68	+0.76 (0.06, 1.45)	0.033^*∗*^
Do you sense a purpose/mission for your life or a reason for being alive?	6.86 ± 2.30	7.48 ± 2.47	+0.62 (−0.10, 1.34)	0.089
How hopeful do you feel?	7.69 ± 1.61	8.28 ± 1.69	+0.59 (0.15, 1.02)	0.010^*∗*^

Data are presented as mean ± standard deviation, and changes are expressed as mean differences for participants that completed both baseline and postintervention assessment visits. Data were obtained using the Quality of Life Patient/Cancer Survivor Version (QOL-CSV), including subscales for physical, psychological, social, and spiritual well-being. Select responses from each subscale are presented. For all items, an increase in score indicates an improvement in QOL. ^*∗*^*p* < 0.05.

**Table 5:** Acceptability of intervention in overweight cancer survivors participating in a 6-month behavioral intervention study (*n* = 29).

Survey questions	Responses	% (*n*)
Would you recommend this program to other survivors?	Yes	96.6 (28)
	No	3.4 (1)

How would you rate the program as a whole?	Excellent	72.4 (21)
	Very good	20.7 (6)
	Good	6.9 (2)
	Fair	0.0 (0)
	Poor	0.0 (0)

How would you rate the group educational classes?	Excellent	58.6 (17)
	Very good	34.5 (10)
	Good	3.4 (1)
	Fair	3.4 (1)
	Poor	0.0 (0)

How would you rate the harvesting at the garden?	Excellent	55.2 (16)
	Very good	37.9 (11)
	Good	6.9 (2)
	Fair	0.0 (0)
	Poor	0.0 (0)

How would you rate the individualized coaching (one-on-one with tele-motivational interviewing coaching)?^a^	Excellent	58.8 (10)
	Very good	29.4 (5)
	Good	11.8 (2)
	Fair	0.0 (0)
	Poor	0.0 (0)

Which program activity was most effective for you? Please pick only one.^a^	Group education	55.2 (16)
	Harvesting produce	34.5 (10)
	Health coaching	17.6 (3)

Did the program impact your overall health in a positive manner?	Yes	96.6 (28)
	No	3.4 (1)

Did the program help you to achieve better dietary patterns that more closely align with the cancer survivor recommendations (primarily plant-based, rich in whole grains, fruits, and vegetables and low in sodium, simple sugars, and red/processed meats)?	Yes	96.6 (28)
	No	3.4 (1)

Did the program help you to improve your physical activity patterns to more closely align with the cancer survivor recommendations (150 minutes of moderate physical activity/week or 10,000 steps/day)?	Yes	93.1 (27)
	No	6.9 (2)

Did the program provide you with a sense of community and support?	Yes	93.1 (27)
	No	6.9 (2)

Do you plan to continue to use the information you received as part of the program to make decisions regarding your health?	Yes	100.0 (29)
	No	0.0 (0)

Data are presented as % and *n*. Data presented are from program-specific evaluation questions asked of participants at postintervention assessment visits. ^a^Tele-motivational interviewing coaching percentage based upon those that utilized the coaching.

## Data Availability

The data used to support the findings of this study are available from the corresponding author upon request.

## Supplementary Materials

Supplementary Materials Figure 1: self-reported change in weight (mean kg) in overweight cancer survivors participating in a 6-month behavioral intervention study. Participants weighed themselves daily and logged weights via an online secure web portal. Weekly means were based on the number of participants each week. Group data were shown at education sessions to demonstrate progress and motivate participants. Weights were not logged for week 0 or week 24 due to baseline and postintervention clinic visits.
