# Supplementary material for: Impact of a Tailored Nutrition and Lifestyle Intervention for Overweight Cancer Survivors on Dietary Patterns, Physical Activity, Quality of Life, and Cardiometabolic Profiles
Source: J Oncol. 2019 Nov 21;2019:1503195. doi: 10.1155/2019/1503195 (PMC6906801; doi:10.1155/2019/1503195)
Supplement: Supplementary Materials — Figure 1: self-reported change in weight (mean kg) in overweight cancer survivors participating in a 6-month behavioral intervention study. Participants weighed themselves daily and logged weights via an online secure web portal. Weekly means were based on the number of participants each week. Group data were shown at education sessions to demonstrate progress and motivate participants. Weights were not logged for week 0 or week 24 due to baseline and postintervention clinic visits. [file 1503195.f1.docx]

**Supplementary Figure**


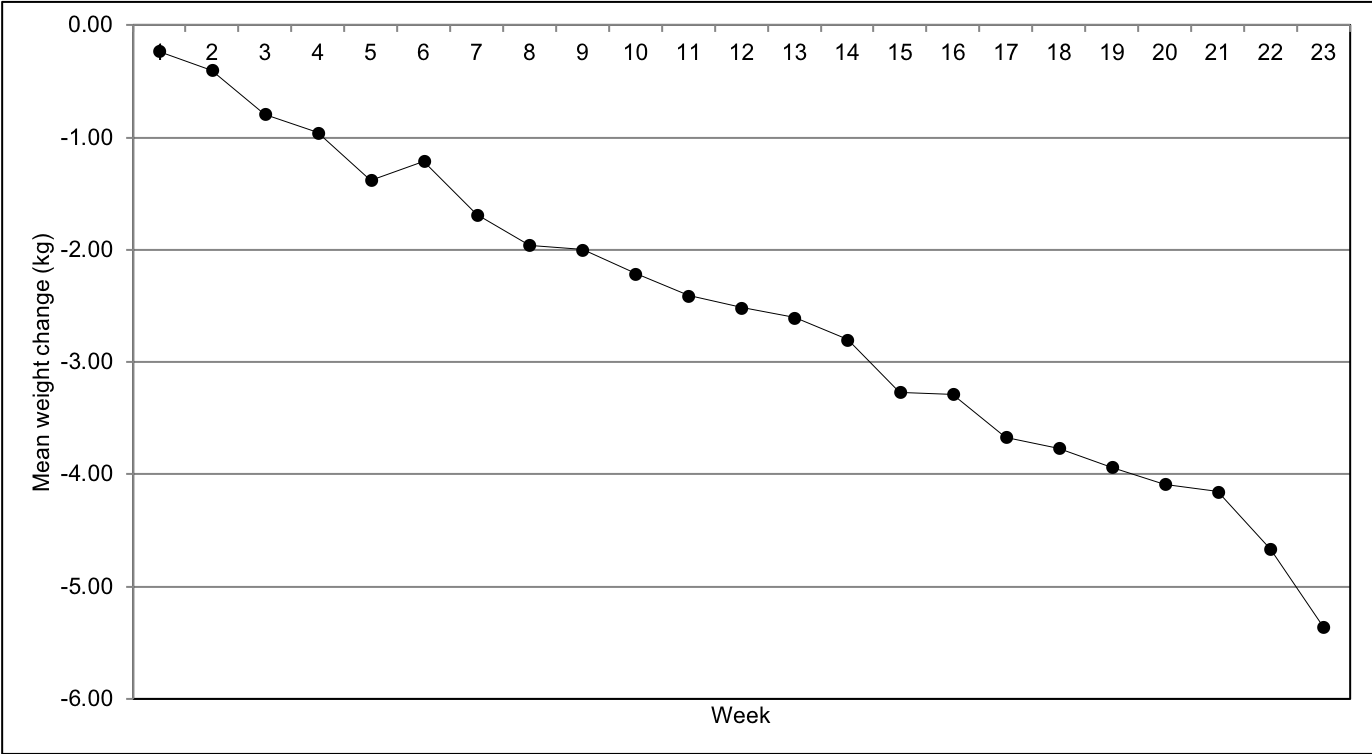
Figure 1: Self-reported Change in Weight (mean kg) in Overweight Cancer Survivors Participating in a 6-month Behavioral Intervention Study. Participants weighed themselves daily and logged weights via an online secure web portal. Weekly means were based on the number of participants each week. Group data were shown at education sessions to demonstrate progress and motivate participants. Weights were not logged for week 0 or week 24 due to baseline and post-intervention clinic visits.
